# GenomicSEM Modelling of Diverse Executive Function GWAS Improves Gene Discovery

**DOI:** 10.1007/s10519-025-10214-4

**Published:** 2025-02-01

**Authors:** Lucas C. Perry, Nicolas Chevalier, Michelle Luciano, CHARGE Consortium

**Affiliations:** School of Philosophy, Psychology and Language Sciences, University of Edinburgh, Edinburgh, UK

## Abstract

Previous research has supported the use of latent variables as the gold-standard in measuring executive function. However, for logistical reasons genome-wide association studies (GWAS) of executive function have largely eschewed latent variables in favour of singular task measures. As low correlations have traditionally been found between individual executive function (EF) tests, it is unclear whether these GWAS have truly been measuring the same construct. In this study, we addressed this question by performing a factor analysis on summary statistics from eleven GWAS of EF taken from five studies, using GenomicSEM. Models demonstrated a bifactor structure consistent with previous research, with factors capturing common EF and working memory- specific variance. Furthermore, the GWAS performed on this model identified 20 new genomic risk loci for common EF and 4 for working memory reaching genome-wide significance beyond what was found in the constituent GWAS, together resulting in 29 newly mapped EF genes. These results help to clarify the underlying genetic structure of EF and support the idea that EF GWAS are capable of measuring genetic variance related to latent EF constructs even when not using factor scores. Furthermore, they demonstrate that GenomicSEM can combine GWAS with divergent and non-ideal measures of the same phenotype to improve statistical power.

**Supplementary Information:**

The online version contains supplementary material available at 10.1007/s10519-025-10214-4.

## Introduction

Executive function (EF) covers the higher-order cognitive processes used to engage in goal-directed behaviour (Ahmed & Miller, 2011, Welsh et al. 2006). It has a role in promoting academic achievement (St Clair-Thompson and Gathercole 2006; Best et al. 2011; Deer et al. 2020) and health behaviours (Reimann et al., 2020), and has been implicated across a wide variety of psychiatric diagnoses including ADHD (Brown 2009; Marije Boonstra et al. 2005), schizophrenia (Johnson, Selfridge & Zalewski, 2001; Wobrock et al. 2009; Orellana and Slachevsky 2013) and depression (Fossati et al. 2002; Letkiewicz et al. 2014; Cotrena et al. 2016). Multiple twin studies have suggested that EF is fully heritable at the latent variable level in adolescents (Friedman et al. 2008; Engelhardt et al. 2015), seemingly violating the supposed ‘law’ of behavioural genetics that no trait is 100% heritable (Plomin and Deary 2015). Although twin studies do support an increased role for environment in predicting EF during adulthood (Friedman et al. 2016; Gustavson et al. 2018; Morrison et al. 2021), they still support additive genetic effects as the primary source of stability in EF over time. Furthermore, twin studies show that genes can explain much of the association between EF and psychiatric diagnoses (Harden et al. 2020), stressful life events (Morrison et al. 2021), and math and reading ability (Daucourt et al., 2020). Thus, understanding the relation between EF and outcomes of interest requires a strong understanding of the role that genes play in EF development.

A key piece of theory underlying our understanding of EF is the unity-diversity model, which is the most well-cited model of EF in the literature (Miyake et al. 2000). This model identifies three separable factors which contribute to EF, inhibition, updating, and shifting, which in addition to their unique variance share common variance known as common EF. The model has been subsequently revised to a bifactor model, consisting of a common EF factor onto which all items load as well as orthogonal updating-specific and shifting-specific factors (Miyake and Friedman 2012; Friedman and Miyake 2017). Notably, this model lacks an inhibition specific factor, as the authors argue there is no inhibition-specific variance. This model has important implications for the measurement of EF, as it has been argued that on a theoretical level, an EF test measuring only EF is impossible. This is because EF is only engaged when directing cognitive resources towards specific tasks, which carry their own cognitive demands that are also captured in task performance (Miyake and Friedman 2012). The need to eliminate this so-called ‘task impurity’, as well as measurement error, has led to the best-practice recommendation that EF be measured by multiple EF tests combined into a latent variable model like unity-diversity through CFA or similar methods (Friedman and Miyake 2017). Importantly, latent EF factors typically outperform individual EF tests in predicting other outcomes of interest (Harden et al. 2020; Gustavson et al. 2022), and produce stronger correlations for EF assessments performed in different contexts (Freis et al., 2023), emphasizing the value of this measurement model. However, there is little-to-no agreement upon a gold-standard set of EF tasks for researchers to use, either when building these factor scores or as single-test measures. A literature review of empirical studies of EF published between 2008 and 2013 identified 109 tasks used for assessment throughout the field, of which 53 were used in multiple studies (Baggetta & Alexander, 2016). The use of such a diverse array of tests might not be an issue if they were highly correlated measures, however this is not the case. Perhaps owing to task impurity, the individual EF tests used in CFAs typically correlated below 0.4, with several showing no significant correlation (Lehto et al. 2003; Miyake et al. 2000). Despite these issues, single-task measurement of EF remains popular in clinical research, a methodological challenge meta-analyses often have to contend with (Andrews et al. 2021; Khoury et al. 2015; Lund et al. 2022; Power et al. 2021). Researchers cannot be sure that correlations with single-test EF measures are actually due to EF, nor that the results will replicate when performed with different EF measures, making the interpretation of any EF study using such measures challenging.

These issues are problematic for genome-wide association studies (GWAS) of EF, where the logistics of the large cohorts required for sufficient statistical power pressures researchers into using abbreviated test batteries that are quicker and easier to administer. As a consequence, marked heterogeneity exists in the approaches to measuring EF phenotypes used in GWAS. Across the 7 GWAS of EF as measured through cognitive tests that could be identified in the literature, only two, trail-marking part B and backwards digit span, were used in more than one study (Ibrahim-Verbaas et al. 2016; Zhang et al. 2018; Donati, Dumontheil, and Meaburn, 2019; Wendel et al., 2021; Hatoum et al. 2023; Dueker et al. 2023; Arnatkeviciute et al. 2023). As these GWAS have largely been performed on separate, weakly-correlated tests rather than latent factors, it is difficult to determine if they are measuring constructs similar to those found elsewhere in EF literature or even in other GWAS. One notable exception is a study conducted in the UK Biobank, which is both the largest GWAS of EF to date (*N* > 400,000) and the only such study to use a factor score (Hatoum et al. 2023). However, the UK Biobank cognitive test battery was not designed to measure EF, with their suitability to do so being based on an analysis of their correlations with common reference EF tests (Fawns-Ritchie & Deary, 2020). The battery included only two traditional EF tasks, the trail-making task and a backwards digit span. As a consequence of these limitations, the factor analysis derived from the UK Biobank’s cognitive tests was only able to capture common EF, and the subsequent polygenic score was not a predictor of the updating or shifting-specific factors (Hatoum et al. 2023). And while the polygenic score was able to predict the common EF factor, its predictive power (β = 0.171, partial *r* =.136) (Hatoum et al. 2023) is still far below what twin studies of EF imply should be predictable from genetics (Friedman et al. 2008; Engelhardt et al. 2015), even considering the limitations of SNP heritability. But larger GWAS meta-analyses cannot be performed with measurement of the phenotype so fractured. Further, measurement error reduces statistical power in GWAS (Liao et al. 2014; van der Sluis et al. 2010), which may account for the failure of EF GWAS not using factor scores to find any significant associations. There is thus good reason to think measuring EF through latent factors will improve the quality of EF GWAS above and beyond what is possible simply by increasing sample size of single-test measures.

GenomicSEM provides a powerful tool to address both the need for latent variables and larger sample sizes in EF GWAS. This method allows for the analysis of multiple GWAS using summary statistics, using them to build a genetic covariance matrix using Linkage Disequilibrium-score regression (LDSC) (Bulik-Sullivan et al. 2015), analogous to the covariance matrix employed by traditional structural equation modelling (Grotzinger et al. 2019). Therefore, rather than requiring building a CFA from multiple EF tests administered to the same participants, GenomicSEM allows a CFA to be built from GWAS of single-test measures without requiring participant overlap. Furthermore, once a model is accepted, GenomicSEM enables users to run a multivariate GWAS on the specified latent factors, using only the summary statistics used to build the model rather than individual-level genetic data. This is accomplished by fitting a structural equation model for each SNP in the analysis, using the SNP effects included in the genetic and sampling covariance matrices to estimate the influence of each SNP on the latent factors (Grotzinger et al. 2019). Thus, EF GWAS ostensibly measuring the same EF component but unsuitable for meta-analysis due to their use of different measures can be combined at the latent factor level, providing an alternative path for increasing sample size while also addressing task impurity. GenomicSEM does not model shared variance due to environment, which is an important limitation of the method compared to traditional SEM. However, this also provides a method of addressing concerns about correlations between tests representing a form of measurement error (Willoughby et al. 2014; Willoughby et al., 2016; Camerota et al. 2020), which should not appear at the genetic level across distinct samples.

The present study represents the first effort to model multiple latent EF factors in GenomicSEM, as well as the first EF model to use GWAS summary statistics taken from different samples. The purpose of the present study will therefore be to examine the factor structure of the currently available EF GWAS, determining the extent to which EF measures from separates studies load onto common constructs. Furthermore, where such common constructs are found, we aim to use GenomicSEM to perform GWAS on the latent factors, to increase statistical power for gene discovery beyond that of the constituent GWAS and allowing for the creation of polygenetic scores of EF factors that should be more predictive of latent EF factors than those derived from the individual studies.

## Method

### Summary Statistics

A literature search was conducted in order to identify GWAS of EF suitable for inclusion in GenomicSEM. Inclusion criteria were EF measured by a cognitive test that showed statistically significant SNP heritability in LDSC (Bulik-Sullivan et al. 2015). As ethnically diverse samples are currently incompatible with GenomicSEM (Grotzinger et al. 2019), samples also needed to be either conducted in a solely European sample or have a European subsample available to meet inclusion criteria. Other demographic features such as age were not part of inclusion criteria. The summary statistics which met inclusion criteria are presented in Table 1. While summary statistics for the Wisconsin Card Sorting Test (Zhang et al. 2018), the PsyCourse Study’s Trail Making Part B and Backwards Digit Span, (Wendel et al., 2021) and the CHARGE consortium’s Trail-Making Part B were also obtained, these failed to show significant SNP heritability in LDSC and so were excluded from further analysis. In addition to their factor score GWAS statistics, Hatoum et al. (2023) made available summary statistics for GWAS of the individual tests that contributed to it. As these tests should in theory tap different EF domains, we used these separate summary statistics when building our model. RsIDs for Arnatkeviciute et al. (2023) were obtained using bedops (Neph et al. 2012). Results for the UK Biobank’s Symbol Digit Substitution Test, the CHARGE consortium’s Stroop test, the Stop-Signal test, and both principal components form the NIHR Bioresource were reverse-coded so all positive loadings would correspond to better task performance.

**Table 1:** GWAS summary statistics for EF test variables used in the present study

Paper	Cohort	Mean Age (years)	Test	EF Component Tapped	Sample Size
Hatoum et al. 2023	UK Biobank	56.85 (8.01)	Trail-Making Part B^a^	Shifting	93,024
			Backwards Digit Span	Working Memory	81,701
			Symbol-Digit Substitution (SDST)	Shifting	84,125
			Pairs Matching	Working Memory or Shifting	81,701
			Prospective Memory	Common EF, possibly Inhibition	162,335
Ibrahim-Verbaas et al. 2016	Cohorts for Heart and Aging Research in Genomic Epidemiology (CHARGE) consortium	55.42 (18.91)	Letter Digit Substitution Test/ Digit Symbol Substitution Task (DSST)	Shifting	32,070
			Stroop	Inhibition	12,866
Donati, Dumontheil, and Meaburn, 2019	Avon Longitudinal Study of Parents and Children (ALSPAC)	10–17 (tasks collected longitudinally)	Working Memory Principal Component^b^	Working Memory, possibly Inhibition	4611
Arnatkeviciute et al. 2023	Meta-analysis (unnamed consortium)	12.05 (2.21)	Stop-Signal Reaction Time (SSRT)	Inhibition	11,715
Rahman et al. 2024	NIHR BioResource	50.48 (14.81)	G6 Principal Component^c^	Common EF, possibly Inhibition and/or Shifting	9879
			G4 Principal Component^d^	Working Memory	9879

^a^ Trail-Making Part B was residualized for Trail Making Part A performance

^b^ The Working Memory Principal Component contained loadings from the N-back, Counting Span, Digit Vigilance, and Dual Tasks

^c^ The G6 Principal Component contained loadings from the Reaction test, Stroop box, Color-word Stroop, Symbol Digit Substitution, Trail making Part A, and Trail making Part B Tasks

^d^ The G4 Principal Component contained loadings from the Working Memory, Matrices, Quiz, and Vocabulary tasks

### Factor Structure

While it was expected that all terms would capture common EF, we additionally assigned each set of GWAS summary statistics loadings onto one of three EF factors- inhibition, working memory, or shifting. Available data were insufficient to distinguish updating from other forms of working memory, so a general working memory factor took the place of updating in our model. While many of the tests presented in Table 1 have long been recognized in the literature as tapping specific EF subcomponents, Hatoum et al.’s (2023) GWAS of the UK Biobank included nonstandard tests for which their capacity to measure EF was established based on correlations with standard EF reference tests (Fawns-Ritchie & Deary, 2020). As the reference tests used tap different EF subcomponents (shifting for Trail Making Part B, working memory for Backwards Digit Span, and complex EF for Tower Test) we used a combination of the relative difference in correlations with these tests as reported in that paper and the demands apparent from the tasks’ design to assign the nonstandard UK Biobank tests to specific EF domains.

Symbol-Digit substitution correlated strongly with the Trails reference tests (*r* = -.558, *p* <.001) and only weakly with Backwards Digit Span (*r* =.177, *p* <.05), indicating that it may contain significant shifting-specific variance (Fawns-Ritchie & Deary, 2020). The Charge consortium also included a similar GWAS combining Letter Digit Substitution and Digit Symbol Substitution, although they used it as a processing speed test (Ibrahim-Verbaas et al. 2016). While this is a common interpretation in the literature, some studies have also supported a relationship between substitution tasks and set shifting, including when measured through Trails B (Jehu et al. 2021; Knowles et al. 2015; Lessov-Schlaggar et al. 2007), which may reflect the need to switch between mental sets of symbol-digit pairings across trials. As such, while we assigned both digit substitution GWAS to set shifting, we also included an orthogonal factor which loaded these GWAS alone, in order to minimize variance due to processing speed captured by our shifting factor.

Pairs matching correlated modestly with Trails B (*r* =.277, *p* <.001) but had no significant correlation with Backwards Digit span (*r* = -.122) suggesting a weaker but still shifting-specific EF component (Fawns-Ritchie & Deary, 2020). This is however surprising, as the task, which involves the participant memorizing an arrangement of cards and then selecting match pairs after they have been flipped over, has apparent working memory demands but no clear link to shifting. As such, we tested models assigning it to both working memory and shifting.

Prospective memory had modest correlations with all EF reference tests (Backwards Digit Span *r* =.265 *p* <.01, Trails B -0.306, *p* <.001) consistent with literature finding prospective memory to be associated with multiple EF domains (Kerns, 2000; Shum et al. 2008; Yang et al. 2011). However, we theorized that the task would be most strongly related to inhibition, which was not represented in the reference tests. The UK Biobank’s prospective memory task is event-based, a classification which previous research has suggested is most strongly related to inhibition (Mahy et al. 2014; Schnitzspahn et al. 2013; Yi et al. 2014; Zuber et al. 2019). Furthermore, the task is conceptually similar to inhibition tasks such as stop-signal and Go/No-Go, both of which are reaction tasks in which a response is inhibited upon receiving a certain signal (Raud et al. 2020). For the prospective memory task, the reaction is to simple onscreen instructions (e.g., ‘touch the blue square’), with the inhibition signal being a previously given set of countermanding instructions (e.g., ‘When you are told to touch the blue square, touch the orange circle’). While the perception of the signal is dependent upon the participant’s prospective memory, responding appropriately to it nevertheless has clear inhibitory demands (Hatoum et al. 2023).

The working memory principal component extracted from the ALSPAC cohort (Donati et al. 2019), while in some ways similar to a CFA approach, comes with issues which require a more nuanced interpretation. PCA is not hypothesis-driven, and analyses all variance in the terms rather than only their shared variance. Consequently, the working memory principal component did not only load onto tasks with obvious working memory demands, but instead had loadings on the Digit Vigilance and Dual Tasks, which are respectively tasks of sustained and divided attention (Donati et al. 2019). In an earlier version of their analysis, the authors also found loadings from the stop-signal task variables at both age 10 and 15 onto their working memory principal component (Donati et al. 2019). While these variables were removed due to their loading onto multiple components, they nevertheless indicated that the principal component they identified as working memory shared variance with the stop-signal task, possibly indicating that it captured variance due to inhibition or some other shared cognitive process. The authors of that paper offered a similar explanation for the principal component they identified as inhibition showing no SNP heritability, suggesting that this may be due to there being no genetic variance unique to inhibition (Donati et al. 2019). Perhaps as a result of these features, during our preliminary analysis with the Stop-Signal Task included, the Stop-Signal, ALSPAC working memory component, and prospective memory showed a relationship which was inconsistent with inhibition or common EF (see supplemental materials). As such, when the decision was made to remove the Stop-Signal, we hypothesized that ALSPAC working memory and prospective memory would retain some relationship. We accounted for this through a correlated error term, declining to assign an interpretation without sufficient conceptual clarity, and retained it in the model as it was found to improve fit.

Finally, the NIHR Bioresource study also included two principal components with potential EF demands (Rahman et al. 2024). While these were conceptualised by the paper as measures of *g*, their use of rotation to produce two factors is outside the norm of *g* research, where *g* is typically defined as the first unrotated principal component of a battery of cognitive tests (Plomin and Deary 2015). As these components included loadings from common EF tests, we hypothesized that they may have captured EF variance as well. G6 contained loadings with two types of Stroop, Trails, as well as Symbol Digit Substitution. As such, we hypothesized that this term would contain common EF demands as well as possible inhibition or shifting-specific demands, although due to its multicollinearity with several other terms in LDSC it could not be included in our tested models. G4 contained loadings from vocabulary and fluid intelligence tasks more in line with *g* research, but also a loading from a digit span task they identified as working or numerical memory. As such, we hypothesized that the term would have working memory demands. However, it should be acknowledged that said working memory task had the weakest loading of the four terms comprising G4 (~ 0.17) and consequentially a phenotypic correlation of 0.57 with the G4 principal component. As such, we compared our accepted model to one which dropped the G4 term entirely in order to ensure that the term aligned in its demands with the rest of the model.

### Analysis

Analysis was conducted in GenomicSEM (Grotzinger et al. 2019). Models were tested using the absolute fit thresholds proposed in the original GenomicSEM paper, with CFI values of ≥ 0.95 considered good fit and ≥ 0.90 considered acceptable fit (Kaplan, 2008), and values of SRMR of < 0.05 considered good fit and < 0.10 considered acceptable fit (Bentler, 1995). The genetic covariance matrix was obtained using LDSC (Bulik-Sullivan et al. 2015); genetic correlations are presented in Table 2. Our initial hypothesis was that the data would fit a unity-diversity three-factor model of set shifting, inhibition, and working memory. We tested both the standard version of this model and a version including an orthogonal factor for substitution task -specific variance. We also tested all possible combinations of models merging two EF factors together. Finally, bifactor models, which added an orthogonal common EF factor to the previously described models, were also tested, both including or eliminating inhibition-specific variance.

The accepted model was run through the user-specified GWAS function in GenomicSEM, regressing SNPS onto each of the latent factors. The genetic covariance matrix was smoothed due to significant differences in *n* between samples, and SNPs which showed a difference of more than 1 in Z-score between the pre- and post-smoothed genetic covariance matrices were removed from the results. SNPs that showed a Q-SNP heterogeneity P-value < 5 × 10^− 08^ were also removed.

LDSC (Bulik-Sullivan et al. 2015) was used to obtain intercepts and genetic covariance with other psychiatric phenotypes, including Hatoum et al.’s (2023) full-sample EF, general cognitive ability (taken from the UK Biobank’s verbal-numerical reasoning test), and processing speed (taken from the UK Biobank’s snap game) summary statistics, as well as summary statistics for educational attainment (Okbay et al. 2022) and 9 different disorders taken from the psychiatric genetics consortium (Arnold et al. 2018; Demontis et al. 2023; Forstner et al. 2021; Grove et al. 2019; Howard et al. 2019; Mullins et al. 2021; Trubetskoy et al. 2022; Watson et al. 2019; Yu et al. 2019). In line with the approach taken by Hatoum et al. (2023) variance shared between our EF factors, general cognitive ability (verbal-numerical reasoning test), and processing speed (snap game) was controlled for using multiple regression in genomicSEM, allowing us to examine the correlations between variance unique to our factors and outcomes of interest. GWAS results were entered into FUMA/MAGMA (Watanabe et al. 2017) to obtain functional annotations and gene mappings. These results were compared to the publicly available FUMA/MAGMA results from Hatoum et al.’s (2023) full sample and the two constituent GWAS which produced significant results (CHARGE DSST and NIH G4) so that overlaps could be removed and a list of novel associations obtained. To filter lead and independent significant SNPS, we first removed all significant SNPs with rsIDs matching significant SNPs in the previous EF GWAS, then used the LD files outputted by FUMA to identify and remove any significant SNPs in our GWAS which were in LD (*r*^*2*^ > 0.6) with significant SNPs from the previous EF GWAS. For gene mappings, we likewise removed any genes with gene symbols matching previous EF associations, as well as any genes associated with the previously removed SNPs. For genomic risk loci, we used the GenomicRanges package in R to identity and remove risk loci overlapping those found in previous EF GWAS (Lawrence et al. 2013).

## Results

Table 2 presents genetic correlations for all summary statistics which met inclusion criteria by showing significant SNP heritability in LDSC. With the exception of Stop-Signal, all summary statistics showed multiple significant genetic overlaps with other measures of EF, indicating that they tap a common phenotype. However, the NIHR G6 displayed multicollinearity with two other terms, and was consequentially removed from the analysis.

**Table 2:** Genetic correlation between GWAS summary statistics

	UKB Trails	UKB Pairs	UKB SDST	UKB Digit	UKB *P*. Memory	CHARGE DSST	CHARGE Stroop	ALSPAC WM	Stop-Signal	NIH G6	NIH G4
UKB Trails	0.1141 (0.0082)	**0.7097 (0.0445)**	**0.7133 (0.0466)**	**0.6685 (0.047)**	**0.3358 (0.0569)**	**0.5832 (0.0663)**	**0.3873 (0.1108)**	**0.4237 (0.1151)**	-0.1003 (0.1235)	**0.9472 (0.1063)**	**0.6379 (0.0794)**
UKB Pairs		0.3765 (0.0163)	**0.6571 (0.0409)**	**0.439 (0.0339)**	**0.3331 (0.0461)**	**0.5568 (0.0559)**	**0.2358 (0.0772)**	**0.324 (0.0789)**	0.0614 (0.0945)	**0.8126 (0.0872)**	**0.534 (0.0561)**
UKB SDST			0.1564 (0.0094)	**0.3224 (0.0442)**	**0.2257 (0.0506)**	**0.8745 (0.0717)**	**0.3596 (0.0986)**	**0.3136 (0.1125)**	0.2078 (0.1232)	**1.0443 (0.0999)**	**0.4092 (0.0717)**
UKB Digit				0.1433 (0.0091)	**0.2328 (0.0514)**	**0.3836 (0.0605)**	**0.4496 (0.1077)**	**0.6427 (0.109)**	-0.0349 (0.1317)	**0.629 (0.1062)**	**0.606 (0.0724)**
UKB P. Memory					0.0368 (0.0035)	**0.3206 (0.077)**	**0.2728 (0.1326)**	**0.4875 (0.1459)**	0.1959 (0.1684)	**0.5353 (0.1385)**	**0.6067 (0.0907)**
CHARGE DSST						0.156 (0.0178)	**0.5018 (0.1355)**	**0.3977 (0.1579)**	**0.4738 (0.173)**	**1.1107 (0.1533)**	**0.3564 (0.1022)**
CHARGE Stroop							0.1035 (0.0336)	-0.2293 (0.2989)	-0.3916 (0.301)	0.0503 (0.2526)	0.2171 (0.1597)
ALSPAC WM								0.279 (0.1097)	0.5458 (0.3198)	**0.675 (0.2609)**	**0.4308 (0.1842)**
Stop-Signal									0.0489 (0.0272)	0.0534 (0.2997)	-0.2009 (0.2166)
NIH G6										0.1388 (0.0552)	**0.6175 (0.2003)**
NIH G4											0.3436 (0.0597)

SNP heritabilities are presented along the diagonal. **Bolded** correlations are significant at *p* <.05. Standard deviations in parentheses. UKB = UK Biobank, Trails = Trail-Making Part B, SDST = Symbol Digit Substitution, Digit = Backwards Digit Span, P. Memory = Prospective Memory, DSST = Digit Symbol Substitution, WM = Working Memory Principal Component

### Confirmatory Factor Analysis

Fit statistics for our CFAs are presented in Table 3. During modelling, the Stop-Signal Reaction Time measure failed to significantly load onto Common EF in bifactor formulations, or inhibition in correlated-factors formulations (see supplemental material). As it also did not significantly correlate with any other measure except for the CHARGE consortium’s DSST, it is excluded from all models presented here.

**Fig. 1:**
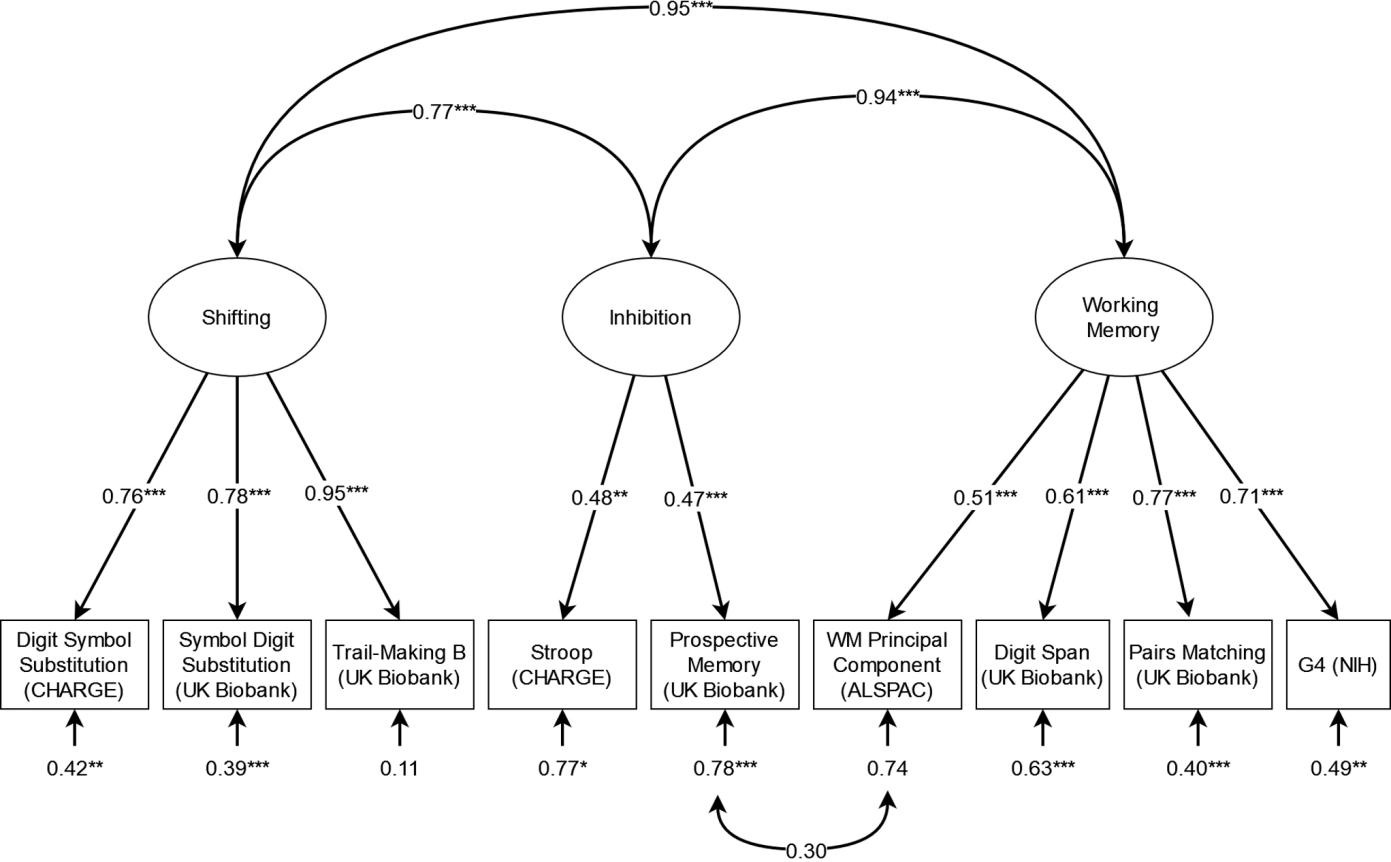
Standardised results for the initial three-factor confirmatory factor analysis model of EF GWAS. *p <.05, ** *p* <.01, *** *p* <.001

**Fig. 2:**
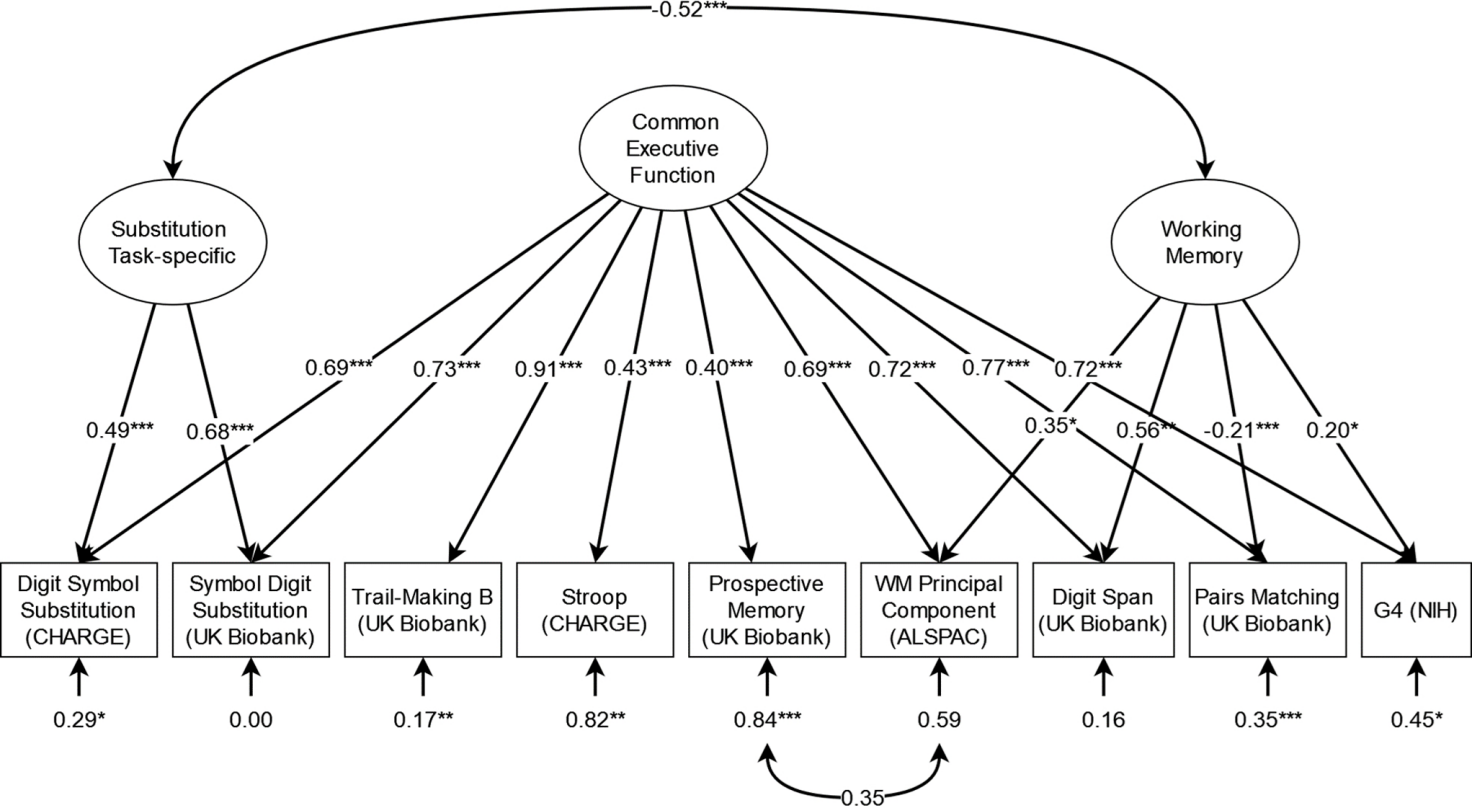
Standardised results for the accepted confirmatory factor analysis model of EF GWAS. *p <.05, ** *p* <.01, *** *p* <.001

The basic three-factor model met acceptable fit thresholds by CFI but failed to do so for SRMR (see Fig. 1). Adding the orthogonal substitution-specific factor slightly improved fit, although it remained below acceptable thresholds (this model also required the residual variance for the CHARGE DSST to be fixed to 0 to avoid empirical under-identification, as no EF terms correlated with the substitution factor). Fit was likewise improved slightly by the bifactor model with working memory, although this, too, fell below acceptable thresholds. After the addition of the orthogonal substitution-specific factor (see Fig. 2), the resulting model fit improved significantly, resulting in good fit by CFI and acceptable fit by SRMR, as well as a statistically significant improvement over both the common factor and previous bifactor model by χ2 and AIC.

Using this adequate-fitting model as a basis, we tested several modifications to confirm our model decisions. We tested the possibility that the substitution factor might instead be characterized as shifting with a model loading Trail Making onto said factor as well. However, while model fit was similar, we found Trail Making loaded only weakly (~ 0.16) and nonsignificantly onto the substitution factor, making its identification with shifting dubious and giving us clear reason to prefer the more parsimonious model without this loading. Given that Pairs matching unexpectedly correlated negatively with working memory, we tested the possibility that it contained only common EF or shifting variance by dropping it from working memory, but found this worsened fit by χ2 and AIC. We also tested a model where prospective memory was assigned to the working memory factor instead of being given a correlated residual with ALSPAC Working Memory, but found this, too, worsened fit. Finally, we tested a model which attempted to account for the substitution factor with a correlated residual, but found that while it was an improvement in fit compared to the bifactor model without the substitution factor, it was still a worse fit than the model with that factor. As none of our modifications were found to improve fit, the bifactor model with working memory and substitution-specific variance was accepted and used for the GWAS.

**Table 3:** Confirmatory factor analysis model results for the genomic structure of EF

Model	χ^2^	df	χ^2^*p*-value	AIC	CFI	SRMR
Common Factor Model	236.1202	27	2.776111e-35	272.1202	0.895631	0.1306878
Three Factor correlated	219.4508	23	5.467484e-34	263.4508	0.9019541	0.1246057
Three factor correlated with Substitution	158.1513	20	1.706908e-23	208.1513	0.9310506	0.1131072
Bifactor with Working Memory	186.1265	22	5.753337e-28	232.1265	0.9180867	0.1081619
*Bifactor With Working Memory and Substitution*	*47.0705*	*19*	*0.0003489703*	*99.0705*	*0.9859904*	*0.09571648*
Bifactor with WM, Substitution is Shifting	47.11906	19	0.0003434276	99.11906	0.9859662	0.1049589
Bifactor drop Pairs from WM	75.56783	20	2.190229e-08	125.5678	0.9722669	0.09663167
Bifactor, Prospective loads onto WM	64.11958	20	1.610782e-06	114.1196	0.9779805	0.104505
Bifactor with WM, Substitution is a correlated residual	129.9266	21	1.047574e-17	177.9266	0.9456362	0.09716726

Endorsed model in italics

As some question remained about the inclusion of the NIH G4 principal component in our model, we fit the accepted model without that term which produced similar fit (CFI = 0.995, SRMR = 0.090). As the resulting summary statistics of the GWAS of the models with and without NIH G4 were at near-to-complete unity in LDSC (0.993 ± 0.048 for common EF and 1.003 ± 0.083 for working memory) we chose to focus on the more powerful GWAS including the NIH G4 principal component.

### GWAS

Table 4 presents a brief summary of our GWAS results after annotation in FUMA. We also present a summary of the subset of our results which were not found in the constituent EF GWAS. Full FUMA and MAGMA results are available in supplementary tables S1 -S20, and include lead and independent significant SNPS, genomic risk loci, identified genes and GWAS Catalog mappings, both with and without overlaps with previous EF GWAS.

**Table 4:** Summary of GWAS results for the common factor model, with and without overlaps with previous EF GWAS

GWAS	Genomic Risk Loci	Lead SNPs	Independent Significant SNPs	Mapped Genes	Top Gene
Common EF	46	47	101	163	*EXOC4*
Novel EF	20	15	27	22	*MON1A*
Working Memory	5	5	5	12	*LRP8*
Novel WM	*4*	4	4	7	*LRP8*

### LDSC

LDSC results for the common EF and working memory-specific factors are presented in Table 5. We also present genetic association results after adjusting for overlap with GWAS of general cognitive ability and processing speed via multiple regression, using summary statistics from Hatoum et al. (2023).

**Table 5:** Genetic relationships between executive function and other cognitive phenotypes

	Genetic Correlations		Multiple Regressions	
	Common EF	Working Memory	Common EF	Working Memory
cEF (UK Biobank)	**0.9645 (0.0462)**	**0.3333 (0.0369)**		
General Cognitive Ability	**0.8572 (0.0358)**	**0.6315 (0.0428)**		
Processing Speed	**0.2459 (0.0277)**	0.0471 (0.0357)		
Educational Attainment	**0.4733 (0.0236)**	**0.4712 (0.0309)**	**-0.3846 (0.1014)**	0.0659 (0.0406)
ADHD	**-0.2689 (0.0302)**	**-0.2093 (0.0445)**	0.1820 (0.1472)	0.0849 (0.0933)
Autism	0.0832 (0.0382)	**0.171 (0.0509)**	-0.3138 (0.1796)	0.0654 (0.0714)
Schizophrenia	**-0.2788 (0.0271)**	0.0338 (0.0357)	*-0.2202 (0.1017)*	**0.2876 (0.0692)**
Bipolar Disorder	**-0.2036 (0.0289)**	*0.0908 (0.0428)*	**-0.4215 (0.1302)**	*0.2348 (0.0778)*
Anxiety	*-0.188 (0.0691)*	0.1396 (0.0931)	-0.4681 (0.2521)	*0.2959 (0.1476)*
Anorexia Nervosa	0.0528 (0.0426)	**-0.2927 (0.0571)**	*0.4966 (0.1918)*	**-0.3808 (0.1028)**
Major Depressive Disorder	**-0.1464 (0.0253)**	-0.011 (0.035)	*-0.2688 (0.1259)*	0.0744 (0.0640)
Obsessive-Compulsive Disorder	-0.0781 (0.0622)	*0.2096 (0.0891)*	-0.3555 (0.2581)	*0.3073 (0.1542)*
Tourette Syndrome	0.0027 (0.0458)	*0.1694 (0.0629)*	0.1236 (0.2081)	*0.3371 (0.1129)*

Multiple regression columns control for overlap with general cognitive ability and processing speed. Bolded results are statistically significant after adjusting for multiple testing (Bonferroni’s corrected *p* <.00192). Italicized results are nominally significant at *p* <.05. Standard deviations in parentheses

## Discussion

The purpose of this study was to perform a confirmatory factor analysis on currently available summary statistics from GWAS of executive function, in order to determine if these studies have been measuring the same construct and allowing us to perform more powerful GWAS on the basis of these shared latent factors. Our analysis produced a bifactor factor model largely in line with previous research, with orthogonal factors for common EF and working-memory-specific variance. That these results could be obtained on the basis of genetic correlations between tests performed largely on unrelated individuals suggests that the unity-diversity model of EF likely captures real biological processes that are reflected in performance on EF tests.

### GWAS

We observed significant results for both common EF and working memory. As expected, given its large contribution to the sample, gene mappings for common EF showed a great deal of unity with the UK Biobank GWAS, including a shared top gene, *EXOC4.* Nevertheless, there were still 22 genes newly associated with EF for this factor, found neither in the UK Biobank nor in any other contributing GWAS. Furthermore, of the 27 novel independent significant SNPs for common EF, 15 were suggestively significant (*p* < 1 * 10^− 6^) in Hatoum et al. (2023). This demonstrates that the inclusion of these outside samples provided increased statistical power and new information, despite contributing only 59,426 additional participants to the GWAS. The top gene of the novel subset was *MON1A*, a gene believed to be involved in protein secretion that has been implicated in a number of other cognitive traits, including occupational attainment (Ko et al. 2022), household income (Hill et al. 2019a, b), intelligence (Hill et al. 2019a, b), creativity (Kim et al. 2024), and schizophrenia (Lam et al. 2019). In FUMA, the common factor also showed significant overlaps with gene-sets derived from GWAS of a number of cognitive phenotypes, most prominently brain morphology, autism, and schizophrenia. Working memory also produced GWAS hits, mapping to 12 genes including 7 not associated with constituent EF GWAS results. The top gene, *LRP8*, has been previously been associated with educational attainment (Lee et al. 2018; Okbay et al., 2022) and various changes in brain morphology (Fan et al. 2022; Naqvi et al. 2021; van der Meer et al. 2021), but interestingly not with any measure of general cognitive ability, suggesting a working memory-specific relationship with these changes may be more proximal.

### LDSC

Our common EF factor was at near-unity with the UK Biobank results in LDSC, showing that they measure the same phenotype. Consequentially, genetic associations are nearly identical to those reported by Hatoum et al. (2023), with differences largely the result of the larger psychiatric genetics consortium studies that have since been made available. However, one noticeable difference is the increase in overlap with cognitive ability from 0.743 (0.013) to 0.857 (0.036). This may be a consequence of the inclusion of the NIH G4 principal component, which was conceived as a measure of *g* (although this term also produced a significant residual in our model, consistent with a partial separability of *g* and common EF). Despite this, associations after controlling for variation shared with cognitive ability and processing speed were of similar magnitude and direction to the UK biobank, although only associations with educational attainment and bipolar disorder survived correction for multiple testing. Associations with bipolar disorder were increased, suggesting that the genetic relationship between common EF and bipolar disorder is mediated by EF-specific genes. However, the association between common EF and educational attainment reversed and became negative. While similar results were reported by Hatoum et al. (2023), the reasons for this finding are unclear. Previous research has suggested a trade-off between common EF and shifting-specific variance, resulting in better shifting being associated with worse outcomes in behavioural domains (Friedman et al. 2011; Herd et al. 2014), but to our knowledge there are no equivalent findings for common EF itself. These findings thus underscore the potential value of research into unique genetic contributions to cognitive phenotypes.

Our working memory factor showed a relatively modest association with the UK Biobank’s common EF but a stronger association with general cognitive ability, consistent with previous findings suggesting that working memory has a relationship to cognitive ability independent of common EF (Friedman et al. 2006). Said relationship appeared to drive the association with educational attainment, ADHD and autism, none of which remained after adjusting for overlap with cognitive ability and processing speed. However, working memory’s significant association with anorexia nervosa increased after the same adjustment, and it gained a significant association with schizophrenia as well as nominally significant associations bipolar disorder and anxiety. Notably, the direction of the effect for these new associations was opposite to that of the common EF, a surprising and difficult-to-interpret finding given that working memory deficits in these disorders are well established (Johnson’Selfridge and Zalewski 2001; Soraggi-Frez et al. 2017; Saldarini et al. 2022). Nevertheless, these results suggest that variance shared with EF plays an important role in these disorders both via the common factor and through factor-specific variance.

### Limitations and Future Directions

Given the potential complexity implied by competing theories of EF, our model contained relatively few terms, constrained by the availability of appropriate GWAS. Our analysis was incapable of separating working memory into updating and maintenance factors, as some EF models do (Engelhardt et al. 2015; Wongupparaj et al. 2015), nor could it separate phonological from visuospatial working memory, which have been shown to have divergent relationships with several psychiatric diagnoses (Perugini et al. 2024; Zilles et al. 2012). One of the more unusual features of our model, the negative loading of pairs matching onto working memory, may be explainable by this distinction, as Pairs is the only visuospatial working memory task in the model. Changes in in the direction of the relationship between cognitive abilities when a common factor is extracted have been demonstrated in previous work (Knyspel & Plomin, 2024). As our bifactor model also extracted common variance (onto which Pairs loaded positively as expected), this may suggest trade-offs between the specific genetic components of visuospatial and phonological working memory. Future GWAS including a range of visuospatial working memory tasks will be important to enable these distinctions to be examined with more clarity.

Our results are unusual with respect to the Stop-Signal test (Arnatkevicuite et al., 2023) which we found had no significant genetic relationship to any other EF test except for the CHARGE consortium’s DSST, and no significant loading onto common EF. While Stop-Signal is a popular measure of inhibitory control, some EF CFAs have reported modest loadings for it (~ 0.15), particularly for adolescent samples (Engelhardt et al. 2015; Freis et al. 2022). And while this may be sufficient to explain the term’s non-significant loading onto common EF, the negative relationship in LDSC between Stop-Signal and Stroop, another popular measure of inhibitory control, might suggest genetic separability beyond the low phenotypic loading. Again, the consortium sample used for the Stop-Signal GWAS skewed towards adolescents, in contrast to the older adult samples used for the UK Biobank and CHARGE, which raises the possibility that the genetic contribution to performance of this task may not be stable across age. Furthermore, if the previous correlation between Stop-Signal and other measures of inhibitory control have been primarily driven by the environment, Genomic SEM, which only takes genetic covariance into account, might present a very different relationship. Friedman et al.’s (2016) results in particular are consistent with this interpretation of our findings, as they found reduced loading for the Stop-Signal task onto inhibition at age 23, along with a loss of any statistically significant role for genetics in predicting its variance. Further research will be needed to disentangle why Stop-Signal produced these divergent results, and what implications this might hold for the task’s use in EF research more broadly.

The loss of Stop-Signal from the model limited our ability to specify an inhibition factor clearly in line with previous research, much less examine any of the proposed subdivisions (Friedman and Miyake 2004; Stahl et al. 2014; Bender et al. 2016; Rey-Mermet et al. 2018). Future GWAS of additional inhibition and shifting tasks may enable the identification of genetic variance unique to these factors. Furthermore, our interpretation of the factors we were able to specify must also be treated with caution. Our summary statistics included GWAS of standard EF tests, nonstandard tests with hypothesized EF demands, and principal components combining both EF and non-EF tests. Although they represent the best GWAS summary statistics currently available for measuring EF, it is nevertheless possible that the latent factors they tap may differ from those found in phenotypic literature. In considering these limitations and how they might be addressed in the future, we in fact highlight a major advantage of CFAs built in GenomicSEM over traditional CFAs. Because the covariance matrix is built on the basis of genetic covariance rather than within-person covariance, terms from future GWAS of EF can be introduced as they become available, collaboratively iterating upon these results and in doing so increasing conceptual clarity.

A final limitation worth noting is the inclusion of GWAS only of European populations in our analysis. This limitation is unfortunately built into GenomicSEM itself, which requires that only GWAS of ethnically homogeneous samples be used. Two of the studies, which contributed GWAS summary statistics to the present study, Ibrahim-Verbaas et al. 2016 and Arnatkeviciute et al. 2023, made a point to construct their GWAS with multi-racial samples. This should be commended, as GWAS have historically and continue to overrepresent European populations in their sample (Popejoy & Fullerton, 2016; Rosenberg et al. 2010). However, because of the limitations of GenomicSEM, we were forced to use only the European subsample of these studies. Members of the GenomicSEM development team have indicated that GenomicSEM will in the future be improved to allow the inclusion of multiple LD files, making trans-ancestry GenomicSEM studies possible (Tucker-Drob, 2023). However, they have also indicated that progress on this development is limited by the paucity of multi-ancestry cohorts.

## Conclusions

This study demonstrates the viability of combining distinct EF tests from differing samples using a latent genetic factor. This will allow for larger meta-analytic GWAS of EF to be conducted in GenomicSEM without the need to unify EF measures across cohorts. However, while this study supports the existence of the unity-diversity structure of EF on the genetic level, the statistical power needed to fully model it for latent factor GWAS still poses a significant challenge at the current stage. Furthermore, our results suggest a need to reexamine the tests used to measure EF, particularly with respect to inhibition. The failure of the Stop-Signal to correlate genetically with other EF tests or load significantly onto latent EF factors limited the models that could be constructed, highlighting a need for greater theoretical clarity as to the relationship between EF tests and the latent construct. GWAS results suggest a potential relationship between various components of EF and psychiatric diagnoses. Furthermore, some results are suggestive of unique associations with specific EF domains, although additional research is needed to clarify these effects. In total, these results suggest that while single-test measures of EF may not be ideal in most circumstances, there is nonetheless value in conducting GWAS of such tests for their contribution to larger analyses of latent genetic factors.

## Electronic Supplementary Material

Below is the link to the electronic supplementary material.

Supplementary Material 1

Supplementary Material 2

## Data Availability

Summary statistics will be made available through GWAS Catalog upon publication. FUMA results will likewise be made publicly available on that platform. Analysis code is available on Github: https://github.com/LCPerry/EFGSEM.

## References

[CR1] Ahmed FS, Stephen Miller L (2011) Executive function mechanisms of theory of mind. J Autism Dev Disord 41(5):667–678. 10.1007/s10803-010-1087-7 20811770 10.1007/s10803-010-1087-7

[CR2] Andrews K, Atkinson L, Harris M, Gonzalez A (2021) Examining the effects of household chaos on child EFs: a meta-analysis. Psychol Bull 147:16–32. 10.1037/bul0000311 33151702 10.1037/bul0000311

[CR83] Arnatkeviciute A, Lemire M, Morrison C, Mooney M, Ryabinin P, Roslin NM, Nikolas M, Coxon J, Tiego J, Hawi Z, Fornito A, Henrik W, Martinot JL, Martinot MLP, Artiges E, Garavan H, Nigg J, Friedman NP, Burton C, … Bellgrove MA (2023) Trans-ancestry meta-analysis of genome wide association studies of inhibitory control. Molecular Psychiatry 1–10. 10.1038/s41380-023-02187-9 10.1038/s41380-023-02187-9 PMC10827666 37500827

[CR4] Arnold PD, Askland KD, Barlassina C, Bellodi L, Bienvenu OJ, Black D, Zai G (2018) Revealing the complex genetic architecture of obsessive-compulsive disorder using meta-analysis. Mol Psychiatry 23(5):1181–1181 28761083 10.1038/mp.2017.154 PMC6660151

[CR5] Baggetta P, Alexander PA, Mind (2016) Brain Educ, 10(1), 10–33. 10.1111/mbe.12100

[CR6] Barendse W (2011) The effect of measurement error of phenotypes on genome wide association studies. BMC Genomics 12(1):232. 10.1186/1471-2164-12-232 21569388 10.1186/1471-2164-12-232 PMC3224145

[CR7] Bender AD, Filmer HL, Garner KG, Naughtin CK, Dux PE (2016) On the relationship between response selection and response inhibition: an individual differences approach. Atten Percept Psychophys 78(8):2420–2432. 10.3758/s13414-016-1158-8 27381631 10.3758/s13414-016-1158-8

[CR201] Bentler PM, Hu LT (1995) Structural Equation Modeling: Concepts, Issues, and Applications 76–99 (SAGE Publications Inc., 1995)

[CR8] Best JR, Miller PH, Naglieri JA (2011) Relations between executive function and academic achievement from ages 5 to 17 in a large, representative national sample. Learn Individual Differences 21(4):327–336. 10.1016/j.lindif.2011.01.007 10.1016/j.lindif.2011.01.007 PMC3155246 21845021

[CR9] Border R, Johnson EC, Evans LM, Smolen A, Berley N, Sullivan PF, Keller MC (2019) No support for historical candidate gene or candidate gene-by-Interaction hypotheses for Major Depression Across multiple large samples. Am J Psychiatry 176(5):376–387. 10.1176/appi.ajp.2018.18070881 30845820 10.1176/appi.ajp.2018.18070881 PMC6548317

[CR10] Brown TE (2009) ADD/ADHD and impaired executive function in clinical practice. Curr Atten Disorders Rep 1(1):37–41. 10.1007/s12618-009-0006-3 10.1007/s11920-008-0065-7 18803914

[CR11] Bulik-Sullivan B, Finucane HK, Anttila V, Gusev A, Day FR, Loh P-R, Duncan L, Perry JRB, Patterson N, Robinson EB, Daly MJ, Price AL, Neale BM (2015) An atlas of genetic correlations across human diseases and traits. Nat Genet 47(11) Article 11. 10.1038/ng.3406 10.1038/ng.3406 PMC4797329 26414676

[CR12] Camerota M, Willoughby MT, Blair CB (2020) Measurement models for studying child executive functioning: questioning the status quo. Dev Psychol 56(12):2236–2245. 10.1037/dev0001127 33104374 10.1037/dev0001127 PMC8284867

[CR13] Cotrena C, Branco LD, Shansis FM, Fonseca RP (2016) Executive function impairments in depression and bipolar disorder: Association with functional impairment and quality of life. J Affect Disord 190:744–753. 10.1016/j.jad.2015.11.007 26606718 10.1016/j.jad.2015.11.007

[CR14] Culverhouse RC, Saccone NL, Horton AC, Ma Y, Anstey KJ, Banaschewski T, Burmeister M, Cohen-Woods S, Etain B, Fisher HL, Goldman N, Guillaume S, Horwood J, Juhasz G, Lester KJ, Mandelli L, Middeldorp CM, Olié E, Villafuerte S, Bierut LJ (2018) Collaborative meta-analysis finds no evidence of a strong interaction between stress and 5-HTTLPR genotype contributing to the development of depression. Mol Psychiatry 23(1):133–142. 10.1038/mp.2017.44 28373689 10.1038/mp.2017.44 PMC5628077

[CR15] Daucourt MC, Haughbrook R, van Bergen E, Hart SA (2020) The association of parent-reported executive functioning, reading, and math is explained by nature, not nurture. Developmental Psychology 56(12):2246. 10.1037/dev0001126 10.1037/dev0001126 PMC8256638 33090832

[CR16] Deer LK, Hastings PD, Hostinar CE (2020) The role of Childhood executive function in explaining Income disparities in Long-Term Academic Achievement. Child Dev 91(5):e1046–e1063. 10.1111/cdev.13383 32712955 10.1111/cdev.13383

[CR17] Demontis D, Walters GB, Athanasiadis G, Walters R, Therrien K, Nielsen TT, Farajzadeh L, Voloudakis G, Bendl J, Zeng B, Zhang W, Grove J, Als TD, Duan J, Satterstrom FK, Bybjerg-Grauholm J, Bækved-Hansen M, Gudmundsson OO, Magnusson SH, Børglum AD (2023) Genome-wide analyses of ADHD identify 27 risk loci, refine the genetic architecture and implicate several cognitive domains. Nat Genet 55(2):198–208. 10.1038/s41588-022-01285-8 36702997 10.1038/s41588-022-01285-8 PMC10914347

[CR18] Donati G, Dumontheil I, Meaburn EL, Mind (2019) Brain Educ, 13(3), 224–233. 10.1111/mbe.12198 10.1111/mbe.12198 PMC6771723 31598132

[CR19] Dueker N, Wang L, Gardener H, Gomez L, Kaur S, Beecham A, Blanton SH, Dong C, Gutierrez J, Cheung YK, Moon YP, Levin B, Wright CB, Elkind MSV, Sacco RL, Rundek T (2023) Genome-wide association study of executive function in a multi-ethnic cohort implicates LINC01362: results from the northern Manhattan study. Neurobiol Aging 123:216–221. 10.1016/j.neurobiolaging.2022.11.016 36658081 10.1016/j.neurobiolaging.2022.11.016 PMC10064578

[CR20] Ecker UKH, Lewandowsky S, Oberauer K, Chee AEH (2010) The components of working memory updating: an experimental decomposition and individual differences. J Experimental Psychology: Learn Memory Cognition 36(1):170–189. 10.1037/a0017891 10.1037/a0017891 20053053

[CR21] Engelhardt LE, Briley DA, Mann FD, Harden KP, Tucker-Drob EM (2015) Genes Unite EFs in Childhood. Psychol Sci 26(8):1151–1163. 10.1177/0956797615577209 26246520 10.1177/0956797615577209 PMC4530525

[CR84] Fan L, Lu M, Qi X, Xin J (2022) Do animations impair executive function in young children? Effects of animation types on the executive function of children aged four to seven years. International Journal of Environmental Research and Public Health 19(15):8962. 10.3390/ijerph19158962 10.3390/ijerph19158962 PMC9332113 35897333

[CR85] Fawns-Ritchie C, Deary IJ (2020) Reliability and validity of the UK Biobank cognitive tests. PLOS ONE 15(4):e0231627. 10.1371/journal.pone.0231627 10.1371/journal.pone.0231627 PMC7170235 32310977

[CR29] Forstner AJ, Awasthi S, Wolf C, Maron E, Erhardt A, Czamara D, Eriksson E, Lavebratt C, Allgulander C, Friedrich N, Becker J, Hecker J, Rambau S, Conrad R, Geiser F, McMahon FJ, Moebus S, Hess T, Buerfent BC, Schumacher J (2021) Genome-wide association study of panic disorder reveals genetic overlap with neuroticism and depression. Mol Psychiatry 26(8):4179–4190. 10.1038/s41380-019-0590-2 31712720 10.1038/s41380-019-0590-2

[CR30] Fossati P, Ergis AM, Allilaire JF (2002) [Executive functioning in unipolar depression: a review. L’Encephale 28(2):97–107 11972136

[CR86] Freis SM, Alexander JD, Anderson JE, Corley RP, De La Vega AI, Gustavson DE, Vrieze SI, Friedman NP (2023) Associations between executive functions assessed in different contexts in a genetically informative sample. Journal of Experimental Psychology: general. 10.1037/xge0001471 10.1037/xge0001471 PMC10843656 37668562

[CR28] Freis SM, Morrison CL, Lessem JM, Hewitt JK, Friedman NP (2022) Genetic and environmental influences on executive functions and intelligence in middle childhood. Dev Sci 25(1):e13150. 10.1111/desc.13150 34288270 10.1111/desc.13150 PMC8639807

[CR22] Friedman NP, Miyake A (2004) The relations among Inhibition and Interference Control functions: a latent-variable analysis. J Exp Psychol Gen 133(1):101–135. 10.1037/0096-3445.133.1.101 14979754 10.1037/0096-3445.133.1.101

[CR23] Friedman NP, Miyake A (2017) Unity and diversity of executive functions: individual differences as a window on cognitive structure. Cortex 86:186–204. 10.1016/j.cortex.2016.04.023 27251123 10.1016/j.cortex.2016.04.023 PMC5104682

[CR25] Friedman NP, Miyake A, Corley RP, Young SE, DeFries JC, Hewitt JK (2006) Not all executive functions are related to intelligence. Psychol Sci 17(2):172–179. 10.1111/j.1467-9280.2006.01681.x 16466426 10.1111/j.1467-9280.2006.01681.x

[CR27] Friedman NP, Miyake A, Young SE, DeFries JC, Corley RP, Hewitt JK (2008) Individual Differences in Executive Functions are almost entirely genetic in origin. J Exp Psychol Gen 137(2):201–225. 10.1037/0096-3445.137.2.201 18473654 10.1037/0096-3445.137.2.201 PMC2762790

[CR26] Friedman NP, Miyake A, Robinson JL, Hewitt JK (2011) Developmental trajectories in toddlers’ self-restraint predict individual differences in executive functions 14 years later: a behavioral genetic analysis. Dev Psychol 47(5):1410–1430. 10.1037/a0023750 21668099 10.1037/a0023750 PMC3168720

[CR24] Friedman NP, Miyake A, Altamirano LJ, Corley RP, Young SE, Rhea SA, Hewitt JK (2016) Stability and change in executive function abilities from late adolescence to early adulthood: a longitudinal twin study. Dev Psychol 52(2):326–340. 10.1037/dev0000075 26619323 10.1037/dev0000075 PMC4821683

[CR31] Gatt JM, Burton KLO, Williams LM, Schofield PR (2015) Specific and common genes implicated across major mental disorders: a review of meta-analysis studies. J Psychiatr Res 60:1–13. 10.1016/j.jpsychires.2014.09.014 25287955 10.1016/j.jpsychires.2014.09.014

[CR35] Grotzinger AD, Rhemtulla M, de Vlaming R, Ritchie SJ, Mallard TT, Hill WD, Ip HF, Marioni RE, McIntosh AM, Deary IJ, Koellinger PD, Harden KP, Nivard MG, Tucker-Drob EM (2019) Genomic structural equation modelling provides insights into the multivariate genetic architecture of complex traits. Nat Hum Behav 3(5). 10.1038/s41562-019-0566-x 10.1038/s41562-019-0566-x PMC6520146 30962613

[CR32] Grove J, Ripke S, Als TD, Mattheisen M, Walters RK, Won H, Pallesen J, Agerbo E, Andreassen OA, Anney R, Awashti S, Belliveau R, Bettella F, Buxbaum JD, Bybjerg-Grauholm J, Bækvad-Hansen M, Cerrato F, Chambert K, Christensen JH, Børglum AD (2019) Identification of common genetic risk variants for autism spectrum disorder. Nat Genet 51(3):431–444. 10.1038/s41588-019-0344-8 30804558 10.1038/s41588-019-0344-8 PMC6454898

[CR33] Gustavson DE, Panizzon MS, Franz CE, Friedman NP, Reynolds CA, Jacobson KC, Xian H, Lyons MJ, Kremen WS (2018) Genetic and environmental architecture of executive functions in midlife. Neuropsychology 32(1):18–30. 10.1037/neu0000389 28857599 10.1037/neu0000389 PMC5814340

[CR34] Gustavson DE, Reynolds CA, Corley RP, Wadsworth SJ, Hewitt JK, Friedman NP (2022) Genetic associations between executive functions and intelligence: a combined twin and adoption study. J Exp Psychol Gen 151(8):1745–1761. 10.1037/xge0001168 34990157 10.1037/xge0001168 PMC9256856

[CR36] Harden KP, Engelhardt LE, Mann FD, Patterson MW, Grotzinger AD, Savicki SL, Thibodeaux ML, Freis SM, Tackett JL, Church JA, Tucker-Drob EM (2020) Genetic associations between executive functions and a general factor of psychopathology. J Am Acad Child Adolesc Psychiatry 59(6):749–758. 10.1016/j.jaac.2019.05.006 31102652 10.1016/j.jaac.2019.05.006 PMC6986791

[CR37] Hatoum AS, Morrison CL, Mitchell EC, Lam M, Benca-Bachman CE, Reineberg AE, Palmer RHC, Evans LM, Keller MC, Friedman NP (2023) Genome-wide Association Study shows that EFing is influenced by GABAergic processes and is a Neurocognitive Genetic Correlate of Psychiatric disorders. Biol Psychiatry 93(1):59–70. 10.1016/j.biopsych.2022.06.034 36150907 10.1016/j.biopsych.2022.06.034 PMC9722603

[CR38] Hedge C, Powell G, Sumner P (2018) The reliability paradox: why robust cognitive tasks do not produce reliable individual differences. Behav Res Methods 50(3):1166–1186. 10.3758/s13428-017-0935-1 28726177 10.3758/s13428-017-0935-1 PMC5990556

[CR39] Herd SA, O׳Reilly RC, Hazy TE, Chatham CH, Brant AM, Friedman NP (2014) A neural network model of individual differences in task switching abilities. Neuropsychologia 62:375–389. 10.1016/j.neuropsychologia.2014.04.014 24791709 10.1016/j.neuropsychologia.2014.04.014 PMC4167201

[CR40] Hill WD, Davies NM, Ritchie SJ, Skene NG, Bryois J, Bell S, Di Angelantonio E, Roberts DJ, Xueyi S, Davies G, Liewald DCM, Porteous DJ, Hayward C, Butterworth AS, McIntosh AM, Gale CR, Deary IJ (2019a) Genome-wide analysis identifies molecular systems and 149 genetic loci associated with income. Nat Commun 10(1):5741. 10.1038/s41467-019-13585-5 31844048 10.1038/s41467-019-13585-5 PMC6915786

[CR41] Hill WD, Marioni RE, Maghzian O, Ritchie SJ, Hagenaars SP, McIntosh AM, Gale CR, Davies G, Deary IJ (2019b) A combined analysis of genetically correlated traits identifies 187 loci and a role for neurogenesis and myelination in intelligence. Mol Psychiatry 24(2):169–181. 10.1038/s41380-017-0001-5 29326435 10.1038/s41380-017-0001-5 PMC6344370

[CR42] Howard DM, Adams MJ, Clarke T-K, Hafferty JD, Gibson J, Shirali M, Coleman JRI, Hagenaars SP, Ward J, Wigmore EM, Alloza C, Shen X, Barbu MC, Xu EY, Whalley HC, Marioni RE, Porteous DJ, Davies G, Deary IJ, McIntosh AM (2019) Genome-wide meta-analysis of depression identifies 102 independent variants and highlights the importance of the prefrontal brain regions. Nat Neurosci 22(3):343–352. 10.1038/s41593-018-0326-7 30718901 10.1038/s41593-018-0326-7 PMC6522363

[CR44] Ibrahim-Verbaas CA, Bressler J, Debette S, Schuur M, Smith AV, Bis JC, Davies G, Trompet S, Smith JA, Wolf C, Chibnik LB, Liu Y, Vitart V, Kirin M, Petrovic K, Polasek O, Zgaga L, Fawns-Ritchie C, Hoffmann P, Mosley TH (2016) GWAS for EF and processing speed suggests involvement of the CADM2 gene. Mol Psychiatry 21(2) Article 2. 10.1038/mp.2015.37 10.1038/mp.2015.37 PMC4722802 25869804

[CR87] Jehu DA, Davis JC, Madden K, Parmar N, Liu-Ambrose T (2021) Minimal clinically important difference of executive function performance in older adults who fall: a secondary analysis of a randomized controlled trial. Gerontology 68(7):771–779. 10.1159/000518939 10.1159/000518939 34657043

[CR45] Johnson’Selfridge M, Zalewski C (2001) Moderator Variables of Executive Functioning in Schizophrenia: Meta-Analytic findings. Schizophr Bull, 27(2) 10.1093/oxfordjournals.schbul.a006876 11354597

[CR200] Kaplan D (2008) Structural equation modeling: foundations and extensions Vol. 10 (Sage, 2008)

[CR89] Kerns KA (2000) The cybercruiser: an investigation of development of prospective memory in children. Journal of the International Neuropsychological Society 6(1):62–70. 10.1017/S1355617700611074 10.1017/s1355617700611074 10761368

[CR90] Khoury JE, Milligan K, Girard TA (2015) Executive functioning in children and adolescents prenatally exposed to alcohol: a meta-analytic review. Neuropsychology Review 25(2):149–170. 10.1007/s11065-015-9289-6 10.1007/s11065-015-9289-6 26037669

[CR48] Kim H, Ahn Y, Yoon J, Jung K, Kim S, Shim I, Park TH, Ko H, Jung S-H, Kim J, Park S, Lee DJ, Choi S, Cha S, Kim B, Cho MY, Cho H, Kim DS, Jang Y, Won H-H (2024) Genome-wide association analyses using machine learning-based phenotyping reveal genetic architecture of occupational creativity and overlap with psychiatric disorders. Psychiatry Res 333:115753. 10.1016/j.psychres.2024.115753 38335777 10.1016/j.psychres.2024.115753

[CR91] Knowles EEM, Weiser M, David AS, Glahn DC, Davidson M, Reichenberg A (2015) The puzzle of processing speed, memory, and executive function impairments in schizophrenia: fitting the pieces together. Biological Psychiatry 78(11):786–793. 10.1016/j.biopsych.2015.01.018 10.1016/j.biopsych.2015.01.018 PMC4547909 25863361

[CR92] Knyspel J, Plomin R (2024) Comparing factor and network models of cognitive abilities using twin data. Intelligence 104:101833. 10.1016/j.intell.2024.101833

[CR49] Ko H, Kim S, Kim K, Jung S-H, Shim I, Cha S, Lee H, Kim B, Yoon J, Ha TH, Kwak S, Kang JM, Lee J-Y, Kim J, Park W-Y, Nho K, Kim DK, Myung W, Won H-H (2022) Genome-wide association study of occupational attainment as a proxy for cognitive reserve. Brain 145(4):1436–1448. 10.1093/brain/awab351 34613391 10.1093/brain/awab351

[CR50] Lam M, Chen C-Y, Li Z, Martin AR, Bryois J, Ma X, Gaspar H, Ikeda M, Benyamin B, Brown BC, Liu R, Zhou W, Guan L, Kamatani Y, Kim S-W, Kubo M, Kusumawardhani A, Liu C-M, Ma H, Huang H (2019) Comparative genetic architectures of schizophrenia in east Asian and European populations. Nat Genet 51(12):1670–1678. 10.1038/s41588-019-0512-x 31740837 10.1038/s41588-019-0512-x PMC6885121

[CR51] Lawrence M, Huber W, Pagès H, Aboyoun P, Carlson M, Gentleman R, Morgan MT, Carey VJ (2013) Software for Computing and Annotating genomic ranges. PLoS Comput Biol 9(8):e1003118. 10.1371/journal.pcbi.1003118 23950696 10.1371/journal.pcbi.1003118 PMC3738458

[CR93] Lee JJ, Wedow R, Okbay A, Kong E, Maghzian O, Zacher M, Nguyen-Viet TA, Bowers P, Sidorenko J, Linnér RK, Fontana MA, Kundu T, Lee C, Li H, Li R, Royer R, Timshel PN, Walters RK, Willoughby EA, … Cesarini D (2018) Gene discovery and polygenic prediction from a 1.1-million-person GWAS of educational attainment. Nature Genetics 50(8):1112–1121. 10.1038/s41588-018-0147-3 10.1038/s41588-018-0147-3 PMC6393768 30038396

[CR52] Lehto JE, Juujärvi P, Kooistra L, Pulkkinen L (2003) Dimensions of EFing: evidence from children. Br J Dev Psychol 21(1):59–80. 10.1348/026151003321164627

[CR94] Lessov-Schlaggar CN, Swan GE, Reed T, Wolf PA, Carmelli D (2007) Longitudinal genetic analysis of executive function in elderly men. Neurobiology of Aging 28(11):1759–1768. 10.1016/j.neurobiolaging.2006.07.018 10.1016/j.neurobiolaging.2006.07.018 16965841

[CR53] Letkiewicz AM, Miller GA, Crocker LD, Warren SL, Infantolino ZP, Mimnaugh KJ, Heller W (2014) Executive function deficits in Daily Life prospectively Predict increases in depressive symptoms. Cogn Therapy Res 38(6):612–620. 10.1007/s10608-014-9629-5 10.1007/s10608-014-9629-5 PMC10544784 37786427

[CR54] Liao J, Li X, Wong T-Y, Wang JJ, Khor CC, Tai ES, Aung T, Teo Y-Y, Cheng C-Y (2014) Impact of Measurement Error on Testing Genetic Association with quantitative traits. PLoS ONE 9(1):e87044. 10.1371/journal.pone.0087044 24475218 10.1371/journal.pone.0087044 PMC3901720

[CR55] Lund JI, Boles K, Radford A, Toombs E, Mushquash CJ (2022) A Systematic Review of Childhood Adversity and EFs outcomes among adults. Arch Clin Neuropsychol 37(6):1118–1132. 10.1093/arclin/acac013 35366302 10.1093/arclin/acac013

[CR95] Mahy CEV, Moses LJ, Kliegel M (2014) The impact of age, ongoing task difficulty, and cue salience on preschoolers’ prospective memory performance: The role of executive function. Journal of Experimental Child Psychology 127:52–64. 10.1016/j.jecp.2014.01.006 10.1016/j.jecp.2014.01.006 24613075

[CR56] Marije Boonstra A, Oosterlaan J, Sergeant JA, Buitelaar JK (2005) Executive functioning in adult ADHD: a meta-analytic review. Psychol Med 35(8):1097–1108. 10.1017/S003329170500499X 16116936 10.1017/s003329170500499x

[CR57] Miyake A, Friedman NP (2012) The Nature and Organization of Individual Differences in Executive Functions: four general conclusions. Curr Dir Psychol Sci 21(1):8–14. 10.1177/0963721411429458 22773897 10.1177/0963721411429458 PMC3388901

[CR58] Miyake A, Friedman NP, Emerson MJ, Witzki AH, Howerter A, Wager TD (2000) The Unity and Diversity of EFs and their contributions to Complex Frontal Lobe tasks: a latent variable analysis. Cogn Psychol 41(1):49–100. 10.1006/cogp.1999.0734 10945922 10.1006/cogp.1999.0734

[CR59] Morrison CL, Rhee SH, Smolker HR, Corley RP, Hewitt JK, Friedman NP (2021) Genetic and environmental influences on stressful life events and their associations with Executive functions in Young Adulthood: a longitudinal twin analysis. Behav Genet 51(1):30–44. 10.1007/s10519-020-10017-9 32959091 10.1007/s10519-020-10017-9 PMC7855628

[CR60] Mullins N, Forstner AJ, O’Connell KS, Coombes B, Coleman JRI, Qiao Z, Als TD, Bigdeli TB, Børte S, Bryois J, Charney AW, Drange OK, Gandal MJ, Hagenaars SP, Ikeda M, Kamitaki N, Kim M, Krebs K, Panagiotaropoulou G, Andreassen OA (2021) Genome-wide association study of more than 40,000 bipolar disorder cases provides new insights into the underlying biology. Nat Genet 53(6):817–829. 10.1038/s41588-021-00857-4 34002096 10.1038/s41588-021-00857-4 PMC8192451

[CR61] Murray BG, Davies DA, Molder JJ, Howland JG (2017) Maternal immune activation during pregnancy in rats impairs working memory capacity of the offspring. Neurobiol Learn Mem 141:150–156. 10.1016/j.nlm.2017.04.005 28434949 10.1016/j.nlm.2017.04.005

[CR96] Naqvi S, Sleyp Y, Hoskens H, Indencleef K, Spence JP, Bruffaerts R, Radwan A, Eller RJ, Richmond S, Shriver MD, Shaffer JR, Weinberg SM, Walsh S, Thompson J, Pritchard JK, Sunaert S, Peeters H, Wysocka J, Claes P (2021) Shared heritability of human face and brain shape. Nature Genetics 53(6):830–839. 10.1038/s41588-021-00827-w 10.1038/s41588-021-00827-w PMC8232039 33821002

[CR62] Neph S, Kuehn MS, Reynolds AP, Haugen E, Thurman RE, Johnson AK, Rynes E, Maurano MT, Vierstra J, Thomas S, Sandstrom R, Humbert R, Stamatoyannopoulos JA (2012) BEDOPS: high-performance genomic feature operations. Bioinformatics 28(14):1919–1920. 10.1093/bioinformatics/bts277 22576172 10.1093/bioinformatics/bts277 PMC3389768

[CR97] Okbay A, Wu Y, Wang N, Jayashankar H, Bennett M, Nehzati SM, Sidorenko J, Kweon H, Goldman G, Gjorgjieva T, Jiang Y, Hicks B, Tian C, Hinds DA, Ahlskog R, Magnusson PKE, Oskarsson S, Hayward C, Campbell A, … Young AI (2022) Polygenic prediction of educational attainment within and between families from genome-wide association analyses in 3 million individuals. Nature Genetics 54(4), Article 4. 10.1038/s41588-022-01016-z 10.1038/s41588-022-01016-z PMC9005349 35361970

[CR63] Orellana G, Slachevsky A (2013) Executive functioning in Schizophrenia. Front Psychiatry. 4https://www.frontiersin.org/articles/10.3389/fpsyt.2013.00035 23805107 10.3389/fpsyt.2013.00035 PMC3690455

[CR98] Perugini A, Fontanillas P, Gordon SD, Fisher SE, Martin NG, Bates TC, Luciano M (2024) Dyslexia polygenic scores show heightened prediction of verbal working memory and arithmetic. Scientific Studies of Reading 1–15. 10.1080/10888438.2024.2365697

[CR64] Plomin R, Deary IJ (2015) Genetics and intelligence differences: five special findings. Mol Psychiatry 20(1). 10.1038/mp.2014.105 10.1038/mp.2014.105 PMC4270739 25224258

[CR99] Popejoy AB, Fullerton SM (2016) Genomics is failing on diversity. Nature 538(7624) Article 7624. 10.1038/538161a 10.1038/538161a PMC5089703 27734877

[CR65] Power J, van IJzendoorn M, Lewis AJ, Chen W, Galbally M (2021) Maternal perinatal depression and child EF: a systematic review and meta-analysis. J Affect Disord 291:218–234. 10.1016/j.jad.2021.05.003 34049191 10.1016/j.jad.2021.05.003

[CR66] Rahman MS, Harrison E, Biggs H, Seikus C, Elliott P, Breen G, Kingston N, Bradley JR, Hill SM, Tom BDM, Chinnery PF (2024) Dynamics of cognitive variability with age and its genetic underpinning in NIHR BioResource genes and cognition cohort participants. Nat Med 1–10. 10.1038/s41591-024-02960-5 10.1038/s41591-024-02960-5 PMC11186791 38745010

[CR100] Raud L, Westerhausen R, Dooley N, Huster RJ (2020) Differences in unity: the go/no-go and stop signal tasks rely on different mechanisms. NeuroImage 210:116582. 10.1016/j.neuroimage.2020.116582 10.1016/j.neuroimage.2020.116582 31987997

[CR101] Reimann Z, Miller JR, Dahle KM, Hooper AP, Young AM, Goates MC, Magnusson BM, Crandall A (2020) Executive functions and health behaviors associated with the leading causes of death in the United States: a systematic review. Journal of Health Psychology 25(2):186–196. 10.1177/1359105318800829 10.1177/1359105318800829 30230381

[CR67] Rey-Mermet A, Gade M, Oberauer K (2018) Should we stop thinking about inhibition? Searching for individual and age differences in inhibition ability. J Experimental Psychology: Learn Memory Cognition 44(4):501–526. 10.1037/xlm0000450 10.1037/xlm0000450 28956944

[CR102] Rosenberg NA, Huang L, Jewett EM, Szpiech ZA, Jankovic I, Boehnke M (2010) Genome-wide association studies in diverse populations. Nature Reviews Genetics 11(5), Article 5. 10.1038/nrg2760 10.1038/nrg2760 PMC3079573 20395969

[CR68] Saldarini F, Gottlieb N, Stokes PRA (2022) Neural correlates of working memory function in euthymic people with bipolar disorder compared to healthy controls: a systematic review and meta-analysis. J Affect Disord 297:610–622 34715175 10.1016/j.jad.2021.10.084

[CR103] Schnitzspahn KM, Stahl C, Zeintl M, Kaller CP, Kliegel M (2013) The role of shifting, updating, and inhibition in prospective memory performance in young and older adults. Developmental Psychology 49(8):1544–1553. 10.1037/a0030579 10.1037/a0030579 23148933

[CR104] Shum D, Cross B, Ford R, Ownsworth T (2008) A developmental investigation of prospective memory: effects of interruption. Child Neuropsychology 14(6):547–561. 10.1080/09297040801947051 10.1080/09297040801947051 18608222

[CR69] Sinopoli VM, Burton CL, Kronenberg S, Arnold PD (2017) A review of the role of serotonin system genes in obsessive-compulsive disorder. Neurosci Biobehavioral Reviews 80:372–381. 10.1016/j.neubiorev.2017.05.029 10.1016/j.neubiorev.2017.05.029 28576508

[CR71] Soraggi-Frez C, Santos FH, Albuquerque PB, Malloy-Diniz LF (2017) Disentangling Working Memory Functioning in Mood States of Bipolar disorder: a systematic review. Front Psychol. 8https://www.frontiersin.org/articles/10.3389/fpsyg.2017.00574 28491042 10.3389/fpsyg.2017.00574 PMC5405335

[CR72] St Clair-Thompson HL, Gathercole SE (2006) Executive functions and achievements in school: shifting, updating, inhibition, and working memory. Q J Experimental Psychol 59(4):745–759. 10.1080/17470210500162854 10.1080/17470210500162854 16707360

[CR70] Stahl C, Voss A, Schmitz F, Nuszbaum M, Tüscher O, Lieb K, Klauer KC (2014) Behavioral components of impulsivity. J Exp Psychol Gen 143(2):850–886. 10.1037/a0033981 23957282 10.1037/a0033981

[CR73] Trubetskoy V, Pardiñas AF, Qi T, Panagiotaropoulou G, Awasthi S, Bigdeli TB, Bryois J, Chen C-Y, Dennison CA, Hall LS, Lam M, Watanabe K, Frei O, Ge T, Harwood JC, Koopmans F, Magnusson S, Richards AL, Sidorenko J, Schizophrenia Working Group of the Psychiatric Genomics Consortium (2022) Mapping genomic loci implicates genes and synaptic biology in schizophrenia. Nature 604(7906):502–508. 10.1038/s41586-022-04434-5 35396580 10.1038/s41586-022-04434-5 PMC9392466

[CR114] Tucker-Drob, E. (2023, May 8). It’s definitely in thepipeline but not coming out soon. With a few notable exceptions, the dataneeded aren’t quite [Comment on the online forum post Multi-AncestryLDSC in GenomicSEM]. Genomic SEM Users Google Group. https://groups.google.com/g/genomic-sem-users/c/uqEy-VX6JuE

[CR105] van der Meer D, Kaufmann T, Shadrin AA, Makowski C, Frei O, Roelfs D, Monereo-Sánchez J, Linden DEJ, Rokicki J, Alnæs D, de Leeuw C, Thompson WK, Loughnan R, Fan CC, Westlye LT, Andreassen OA, Dale AM (2021) The genetic architecture of human cortical folding. Science Advances 7(51): eabj9446. 10.1126/sciadv.abj9446 10.1126/sciadv.abj9446 PMC8673767 34910505

[CR74] van der Sluis S, Verhage M, Posthuma D, Dolan CV (2010) Phenotypic complexity, Measurement Bias, and poor phenotypic resolution contribute to the Missing Heritability Problem in Genetic Association Studies. PLoS ONE 5(11):e13929. 10.1371/journal.pone.0013929 21085666 10.1371/journal.pone.0013929 PMC2978099

[CR106] Watanabe K, Taskesen E, van Bochoven A, Posthuma D (2017) Functional mapping and annotation of genetic associations with FUMA. Nature Communications 8(1), Article 1. 10.1038/s41467-017-01261-5 10.1038/s41467-017-01261-5 PMC5705698 29184056

[CR75] Watson HJ, Yilmaz Z, Thornton LM, Hübel C, Coleman JRI, Gaspar HA, Bryois J, Hinney A, Leppä VM, Mattheisen M, Medland SE, Ripke S, Yao S, Giusti-Rodríguez P, Hanscombe KB, Purves KL, Adan RAH, Bulik CM, Eating Disorders Working Group of the Psychiatric Genomics Consortium (2019) Genome-wide association study identifies eight risk loci and implicates metabo-psychiatric origins for anorexia nervosa. Nat Genet 51(8):1207–1214. 10.1038/s41588-019-0439-2 31308545 10.1038/s41588-019-0439-2 PMC6779477

[CR107] Welsh, M. C., Friedman, S. L., & Spieker, S. J. (2006).Executive Functions in Developing Children: Current Conceptualizations andQuestions for the Future. In Blackwell handbook of early childhooddevelopment (pp. 167–187). Blackwell Publishing. 10.1002/9780470757703.ch9

[CR108] Wendel B, Papiol S, Andlauer TFM, Zimmermann J, Wiltfang J, Spitzer C, Senner F, Schulte EC, Schmauß M, Schaupp SK, Repple J, Reininghaus E, Reimer J, Reich-Erkelenz D, Opel N, Nenadić I, Meinert S, Konrad C, Klöhn-Saghatolislam F, … Heilbronner U (2021) A genome-wide association study of the longitudinal course of executive functions. Translational Psychiatry 11(1), Article 1. 10.1038/s41398-021-01510-8 10.1038/s41398-021-01510-8 PMC8272719 34247186

[CR77] Willoughby M, Holochwost SJ, Blanton ZE, Blair CB (2014) EFs: formative Versus Reflective Measurement. Measurement: Interdisciplinary Res Perspect 12(3):69–95. 10.1080/15366367.2014.929453

[CR109] Willoughby MT, Blair CB, The Family Life Project Investigators (2016) Measuring executive function in early childhood: a case for formative measurement. Psychological Assessment 28(3):319–330. 10.1037/pas0000152 10.1037/pas0000152 PMC4695318 26121388

[CR78] Wobrock T, Ecker UKH, Scherk H, Schneider-Axmann T, Falkai P, Gruber O (2009) Cognitive impairment of executive function as a core symptom of schizophrenia. World J Biol Psychiatry 10(4–2):442–451. 10.1080/15622970701849986 18609418 10.1080/15622970701849986

[CR79] Wongupparaj P, Kumari V, Morris RG (2015) The relation between a multicomponent working memory and intelligence: the roles of central executive and short-term storage functions. Intelligence 53:166–180. 10.1016/j.intell.2015.10.007

[CR80] Xu F, Wang B, Yao J (2018) Association between the SLC6A4 gene and schizophrenia: an updated meta-analysis. Neuropsychiatr Dis Treat 15:143–155. 10.2147/NDT.S190563 30643413 10.2147/NDT.S190563 PMC6314053

[CR110] Yang J, Manolio TA, Pasquale LR, Boerwinkle E, Caporaso N, Cunningham JM, de Andrade M, Feenstra B, Feingold E, Hayes MG, Hill WG, Landi MT, Alonso A, Lettre G, Lin P, Ling H, Q1 Q2 Lowe W, Mathias RA, Melbye M, … Visscher PM (2011) Genome partitioning of genetic variation for complex traits using common SNPs. Nature Genetics 43(6):519–525. 10.1038/ng.823 10.1038/ng.823 PMC4295936 21552263

[CR111] Yi L, Fan Y, Joseph L, Huang D, Wang X, Li J, Zou X (2014) Event-based prospective memory in children with autism spectrum disorder: the role of executive function. Research in Autism Spectrum Disorders 8(6):654–660. 10.1016/j.rasd.2014.03.005

[CR81] Yu D, Sul JH, Tsetsos F, Nawaz MS, Huang AY, Zelaya I, Illmann C, Osiecki L, Darrow SM, Hirschtritt ME, Greenberg E, Muller-Vahl KR, Stuhrmann M, Dion Y, Rouleau G, Aschauer H, Stamenkovic M, Schlögelhofer M, Sandor P, the Psychiatric Genomics Consortium Tourette Syndrome Working Group (2019) Interrogating the genetic determinants of Tourette’s syndrome and other Tic disorders through genome-wide Association studies. Am J Psychiatry 176(3):217–227. 10.1176/appi.ajp.2018.18070857. Tourette Association of America International Consortium for Genetics, the Gilles de la Tourette GWAS Replication Initiative, the Tourette International Collaborative Genetics Study 30818990 10.1176/appi.ajp.2018.18070857 PMC6677250

[CR82] Zhang H, Zhou H, Lencz T, Farrer LA, Kranzler HR, Gelernter J (2018) Genome-wide association study of cognitive flexibility assessed by the Wisconsin Card sorting test. Am J Med Genet Part B: Neuropsychiatric Genet 177(5):511–519. 10.1002/ajmg.b.32642 10.1002/ajmg.b.32642 PMC6110090 30134085

[CR112] Zilles D, Meyer J, Schneider-Axmann T, Ekawardhani S, Gruber E, Falkai P, Gruber O (2012) Genetic polymorphisms of 5-HTT and DAT but not COMT differentially affect verbal and visuospatial working memory functioning. European Archives of Psychiatry and Clinical Neuroscience 262(8): 667–676. 10.1007/s00406-012-0312-0 10.1007/s00406-012-0312-0 PMC3491187 22454241

[CR113] Zuber S, Mahy CEV, Kliegel M (2019) How executive functions are associated with event-based and time-based prospective memory during childhood. Cognitive Development 50:66–79. 10.1016/j.cogdev.2019.03.001

